# Simultaneous transcatheter replacement of four cardiac valves in a 58-year-old female

**DOI:** 10.1093/eurheartj/ehaf757

**Published:** 2025-09-25

**Authors:** Changdong Zhang, Mei Liu, Xiaoke Shang

**Affiliations:** Department of Cardiovascular Surgery, Union Hospital, Tongji Medical College, Huazhong University of Science and Technology, No.1277 Jiefang Avenue, WuHan City, Hubei Province 430022, China; Clinical Nutrition Department, Wuhan No.1 Hospital, No. 215 Zhongshan Avenue, WuHan City, Hubei Province 430022, China; Department of Cardiovascular Surgery, Union Hospital, Tongji Medical College, Huazhong University of Science and Technology, No.1277 Jiefang Avenue, WuHan City, Hubei Province 430022, China

**Figure:**
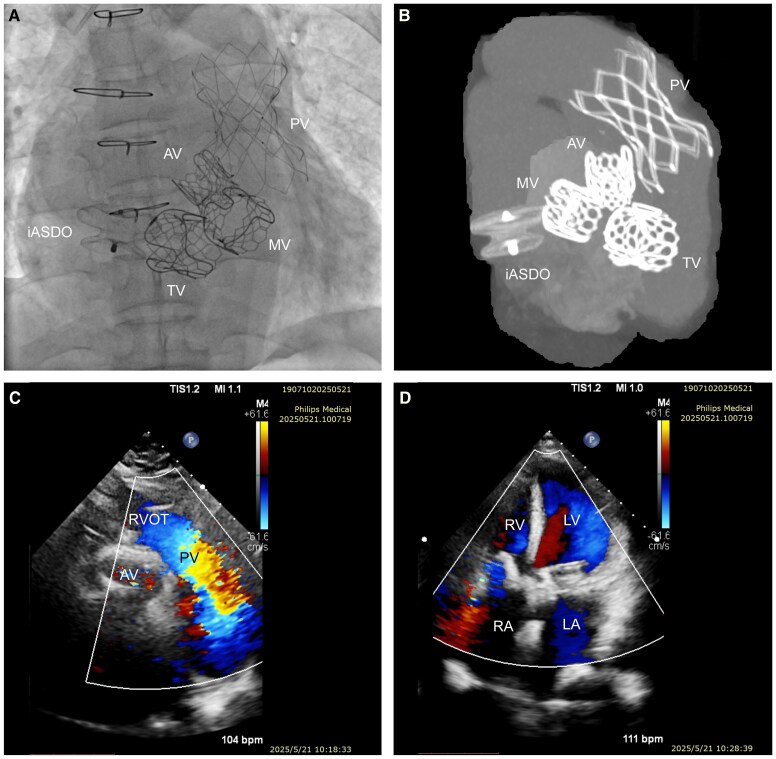


A 58-year-old woman with rheumatic heart disease and triple bioprosthetic valve replacement (aortic, mitral, tricuspid; 2011) presented with one year of worsening dyspnoea, chest tightness, and palpitations. Thirteen years after surgery, she exhibited NYHA III heart failure, irregular rhythm, and biphasic murmur. Echocardiography revealed advanced degeneration with severe stenosis and regurgitation of all prostheses, pulmonary valve regurgitation, right atrial thrombus (4.4 × 2.8 cm), biatrial enlargement, and biventricular dysfunction (LVEF 32%, RVEF 19%, FAC 26%). A fully percutaneous procedure was performed, sequentially deploying a balloon expandable PrizValve 23# in the aortic, PrizValve 26# in the mitral, PrizValve 29# in the tricuspid, and a self-expanding PT-Valve 40/26 in the pulmonary position. An iatrogenic atrial septal defect created during the procedure was closed to prevent paradoxical embolism (*Panels A* and *B*). Postoperatively, warfarin maintained the international normalized ratio between 2 and 3. At 15 months, all valves functioned well with thrombus resolution (*Panels C* and *D*). This is the first reported case of simultaneous transcatheter replacement of all four valves.

*Panel A*: Immediate postoperative fluoroscopy demonstrated four transcatheter valves and closure of an iatrogenic atrial septal defect with an occluder. *Panel B*: CT after implantation of four valves and iatrogenic atrial septal defect closure. *Panel C*: Echocardiographic short axis view of the great arteries demonstrated no regurgitation of the implanted aortic and pulmonary valves and no flow acceleration across the pulmonary valve. *Panel D*: Echocardiographic apical four chamber view demonstrated no regurgitation of the implanted mitral and tricuspid valves.

Supplementary data are not available at *European Heart Journal* online.

All authors declare no disclosure of interest for this contribution.

The data that support the findings of this study are available from the corresponding author upon reasonable request.

No funding.

